# Cardiac thrombus in acute ischemic stroke: impact on endovascular thrombectomy utilization

**DOI:** 10.1186/s12959-024-00650-3

**Published:** 2024-09-27

**Authors:** Zafar Ali, Abdul Wali Khan, Islam Shatla, Sayyeda Aleena Mufarrih, Rithvik Talluri, Talal Asif

**Affiliations:** 1 University of Kansas Medical Center, Kansas City, KS USA; 2 Dept of Internal Medicine, University of Missouri Kansas City School of Medicine, Kansas City, MO USA; 3 Khyber Girls Medical College, Peshawar, Pakistan; 4 Dept of Cardiology, University of Missouri Kansas City School of Medicine, Kansas City, MO USA

**Keywords:** Cardiac embolism, Endovascular thrombectomy, Acute ischemic stroke

## Abstract

Cardiac embolism plays a very significant role in acute ischemic strokes (AIS), constituting approximately one-third of cases. The origin of these emboli often stems from intracardiac thrombi in the left atrium or left ventricle. Utilizing the National Readmission Database from 2016 to 2019, we investigate the prevalence of cardiac thrombi in AIS patients and explore their potential correlation with endovascular thrombectomy (EVT) utilization, and mortality rates. Among 1,272,456 AIS patients, 0.6% had concurrent cardiac thrombus, with an increasing trend observed over the study period (P value < 0.001). The cardiac thrombus cohort showed a higher prevalence of comorbidities such as congestive heart failure and atrial fibrillation. After propensity-score matching, groups were well-balanced in terms of baseline characteristics. Patients within the cardiac thrombus cohort experienced a longer hospital stay (median 5 days vs. 3 days), but no significant difference in mortality was noted. Importantly, the cardiac thrombus cohort demonstrated a higher frequency of EVT utilization, suggesting a link to larger vessel occlusions. Despite matching based on atrial fibrillation, the EVT utilization in the cardiac thrombus cohort remained high, highlighting a significant association. While 30-day readmission rates were comparable, cardiac thrombus patients faced a higher risk of gastrointestinal bleeding and hemorrhagic stroke, likely attributed to anticoagulation use. Limitations include potential miscoding in the administrative database and a lack of detailed imaging findings. In conclusion, this study highlights the increased likelihood of EVT in AIS patients with cardiac thrombus, possibly indicative of larger vessel occlusion associated with cardiac thrombus.

## Introduction

Cardiac embolism plays a significant role in the occurrence of acute ischemic stroke (AIS), accounting for roughly one-third of cases [1]. These emboli can be from intracardiac thrombi, which may develop within the left atrium or left ventricle. Left atrial thrombi can originate from the primary left atrium or the atrial appendage, often associated with atrial fibrillation [2]. On the other hand, left ventricular thrombi typically occur following acute myocardial infarction or in patients with a reduced ejection fraction [3]. Atrial fibrillation has been shown to be associated with large strokes requiring endovascular thrombectomy (EVT) [4]. There is limited data regarding the prevalence of cardiac thrombi among AIS patients and its potential association with and the likelihood of requiring EVT. This study seeks to address this knowledge gap by investigating the prevalence of cardiac thrombi in AIS patients and determining whether their presence is indicative of increased utilization of EVT and elevated mortality rates.

## Methods

We used the National Readmission Database (NRD) to identify patients who were hospitalized for AIS and had a concurrent cardiac thrombus between 2016 and 2019. The NRD, a publicly accessible database developed by the Agency for Healthcare Research and Quality (AHRQ) as part of the Healthcare Cost and Utilization Project (HCUP) [5]. We used the International Classification of Diseases Tenth Revisions ICD-10) codes for AIS (I63) and cardiac thrombus (I513 and I236). Patients were stratified according to the presence of concurrent cardiac thrombus into two groups: AIS with cardiac thrombus and without cardiac thrombus. An unadjusted analysis was performed to compare baseline characteristics between both groups. Our main objectives were to examine the prevalence of cardiac thrombus in patients hospitalized for AIS and utilization of EVT, as well as to assess mortality and the readmission rate within 30 days. To calculate the 30-day readmission, we excluded patients who died during index hospitalization and those admitted in the month of December.

To account for the non-random selection of patients with inherent differences in characteristics, we matched patients by creating a propensity score using the relevant variables as independent variables. A P-value of < 0.05 was considered statistically significant. We used R (4.3.2) to run the statistical analysis for this study.

## Results

A total of 1,272,456 patients with acute ischemic stroke were identified during our study period, of whom 7545 (0.6%) had cardiac thrombus. The median age (IQR) was 64 years (54,74) for the cardiac thrombus group and 69 years (58,79) for patients without cardiac thrombus. Baseline comorbidities such as congestive heart failure (CHF) (57% vs. 16%, *p* < 0.001), coronary artery disease (54% vs. 27%, *p* < 0.001), non-ischemic cardiomyopathy (20% vs. 3.2%, *p* < 0.001), and atrial fibrillation/flutter (35 vs. 22%, *p* < 0.001) more prevalent in the cardiac thrombus group Table 1.

**Table 1:** Baseline characteristics of the study groups

Variables	Acute ischemic stroke with intracardiac thrombus *N* = 7,545	Acute ischemic stroke without intracardiac thrombus *N* = 1,264,911	*P*-Value
Age (Median)	64 (54,76)	69 (58,79)	< 0.001
Weekend Admission	2,011 (27%)	321,238 (25%)	0.11
Female	2538 (34%)	599,517 (47%)	< 0.001
Diabetes mellitus	1,203 (16%)	226,967 (18%)	0.04
Hypertension	3,192 (42%)	768,792 (61%)	< 0.001
CAD	4,125 (55%)	337,973 (27%)	< 0.001
Atrial flutter	2,644 (35%)	280,042 (22%)	< 0.001
Congestive heart failure	4,315 (57%)	203,640 (16%)	< 0.001
COPD	744 (10.0%)	124,304 (9.8%)	0.8
Coagulopathy	746 (9.9%)	60,918 (4.8%)	< 0.001
Renal failure	1,460 (19%)	211,288 (17%)	< 0.001
Peripheral vascular disease	1,294 (17%)	120,461 (9.5%)	< 0.001
Obesity	1054 (14%)	189,753 (15%)	0.1
Prior Stroke	2,386 (32%)	353,871 (28%)	< 0.001
Primary expected payer			< 0.001
Medicare	3,887 (52%)	761,186 (60%)	
Medicaid	1,238 (16%)	124,364 (9.8%)	
Private insurance	1,741 (23%)	276,239 (22%)	
Self-pay	415 (5.5%)	59,911 (4.7%)	
No Charge	36 (0.5%)	6,904 (0.5%)	
Other	211 (2.8%)	34,270 (2.7%)	
Solid Tumor	317 (4.2%)	40,886 (3.2%)	< 0.002
Metastatic Disease	223 (3.0%)	22,808 (1.8%)	< 0.001
Liver Disease	227 (3.0%)	23,928 (1.9%)	< 0.001

^CAD: Coronary Artery Disease, COPD: Chronic Obstructive Pulmonary Disease^

After propensity-score matching, both groups were well-balanced in terms of baseline characteristics (Table 2).

**Table 2:** Baseline characteristics of matched cohort

Variables	Acute ischemic stroke with intracardiac thrombus *N* = 7,467^1^	Acute ischemic stroke without intracardiac thrombus *N* = 6792	*P*-Value
Age (Median)	64 (54, 74)	65 (56, 76)	0.006
Female	2,504 (34%)	2,195 (32%)	0.2
Diabetes mellitus	1,199 (16%)	1,011 (15%)	0.1
Hypertension	3,163 (42%)	2,865 (42%)	0.5
CAD	4,052 (54%)	3,837 (57%)	0.2
Atrial flutter	2,618 (35%)	2,473 (36%)	0.20
Congestive heart failure	4,272 (57%)	4,059 (60%)	0.12
COPD	744 (10.0%)	124,304 (9.8%)	0.80
Renal failure	1,444 (19%)	1,316 (19%)	0.70
Peripheral vascular disease	1,288 (17%)	1,206 (18%)	0.70
Obesity	1042 (14%)	923 (14%)	0.70
Prior Stroke	2,368 (32%)	2,157 (32%)	0.80
Solid Tumor	313 (4.2%)	269 (4%)	0.70
Metastatic Disease	220 (2.9%)	164 (2.4%)	0.50
Liver Disease	221 (3.0%)	145 (2.1%)	0.05

CAD: Coronary Artery Disease; COPD: Chronic Obstructive Pulmonary Disease

**Fig. 1:**
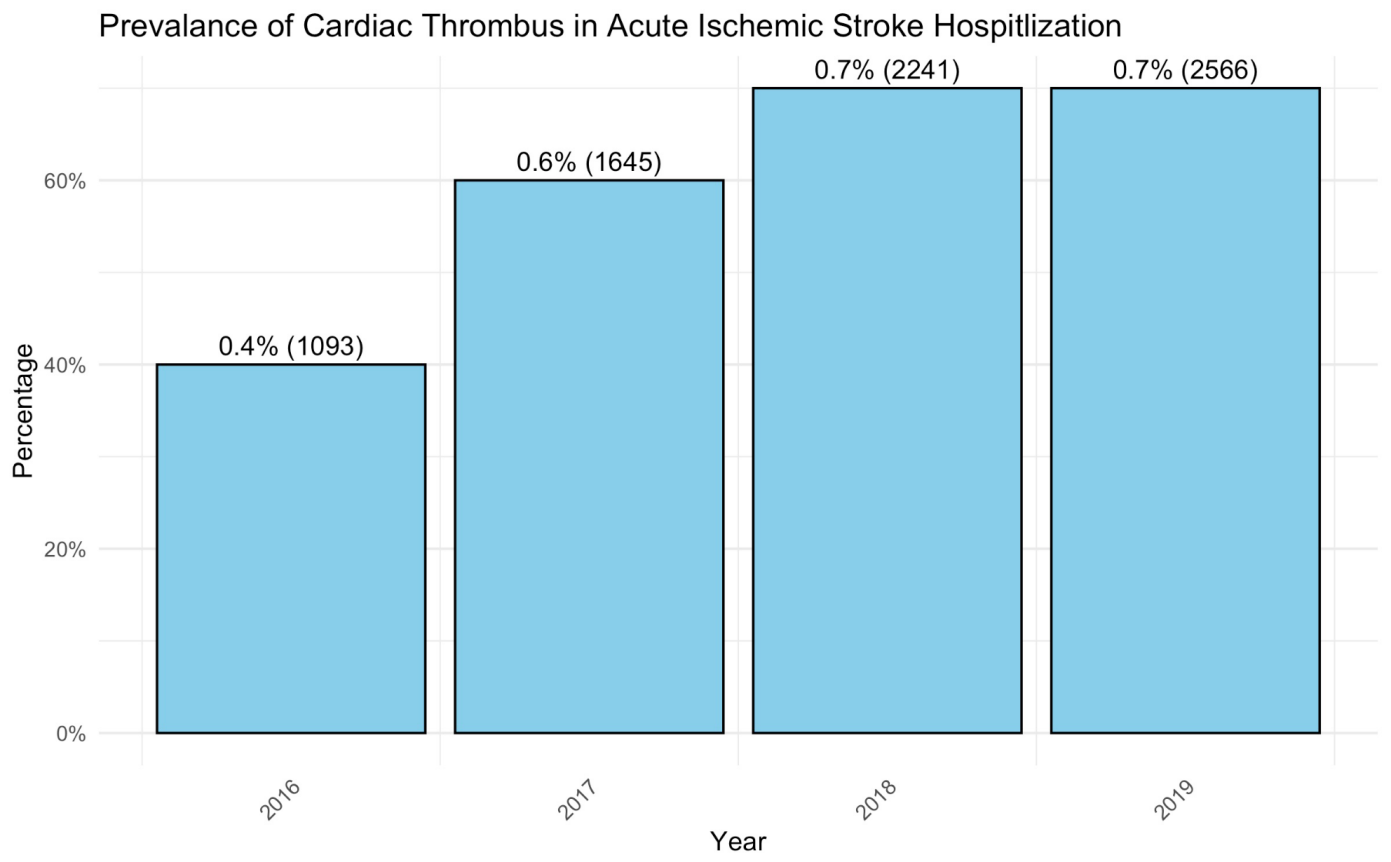
Trends in the prevalence of concurrent cardiac thrombus and acute ischemic

There was an uptrend of cardiac thrombus prevalence from 0.4% in 2016 (1,093 cases) to 0.7% (2,566 cases) during the study period (P value < 0.001) (Fig. 1).

The median length of hospital stay (LOS) for patients with cardiac thrombus was 5 days, whereas it was 3 days for patients without cardiac thrombus (*p* < 0.001 as shown in the table. There was no significant difference in inpatient mortality for index hospitalization between patients with or without cardiac thrombus.

In the matched cohort, there was a higher frequency of advanced interventions, such as EVT (5.6% vs. 3.9%, *p* = 0.004), in the group with cardiac thrombus, while there was no difference in the utilization of tissue plasminogen activator (TPA) or both TPA and EVT between the two groups. The rate of 30-day readmission did not differ between patients with and without cardiac thrombus. However, patients with cardiac thrombus exhibited a higher risk of gastrointestinal bleeding and hemorrhagic stroke, likely attributable to the use of anticoagulation in this group (Table 3).

**Table 3:** In-hospital outcome in patients with AIS and intracardiac thrombus (Matched-Cohort)

Variables	Acute ischemic stroke with intracardiac thrombus *N* = 7,471	Acute ischemic stroke without intracardiac thrombus. *N* = 6,781	*P*-Value
Mortality rate	720 (9.6%)	627 (8.2%)	0.2
EVT	420 (5.6%)	275 (4.1%)	0.003
LOS	5 (3, 10)	3 (2, 7)	< 0.001
TPA	728 (9.7%)	692 (10%)	0.4
Both TPA and EVT	87 (1.2%)	66 (1.0%)	0.3
30-days readmission	759 (11%)	634 (10%)	0.4
Hemorrhagic Stroke Readmission	29 (3.8%)	6 (1.0%)	0.014
GI Bleed Readmit	65 (8.6%)	34 (5.4%)	0.045
Acute Ischemic Stroke Readmission	141 (19%)	133 (21%)	0.4

^EVT: Endovascular Thrombectomy, TPA: Tissue Plasminogen Activator, LOS: Length of Hospital Stay^

## Discussion

To the best of our knowledge, this study represents the first population-based investigation into the relationship between cardiac thrombus and EVT in AIS patients. Our findings reveal that AIS patients with concurrent cardiac thrombus are more likely to undergo EVT. Interestingly, we observed no significant difference in inpatient mortality rates between patients with and without cardiac thrombus. While 30-day readmission rates were comparable between the two groups, patients with cardiac thrombus showed a higher propensity for readmission due to hemorrhagic stroke and gastrointestinal bleeding.

Our findings indicate a higher rate of EVT usage among patients with cardiac thrombus, suggesting this group may be more prone to large vessel occlusions that require advanced interventional techniques. While previous research has linked atrial fibrillation to large vessel occlusions and increased EVT use [4], our study’s results are particularly noteworthy. Even after propensity matching for atrial fibrillation, we observed higher EVT utilization in the cardiac thrombus group. This implies that cardiac thrombus itself may be independently associated with large vessel occlusions, thus necessitating EVT. Our study showed a growing prevalence of cardiac thrombus in AIS patients from 2016 to 2019. This uptrend can be attributed to advancements in diagnostic imaging, particularly the increased utilization of cardiac magnetic resonance imaging (CMR) which enhances the detection of intracardiac thrombi [6]. Another study has showed that one in every 12 patients with AIS had cardiac thrombus detected on cardiac tomography (CT) and that had worse functional outcome [7]. This is likely a call for further investigation if more advanced imagine like CT or CMR will be beneficial for patient with AIS to detect cardiac thrombus and potentially affect management.

Our analysis revealed no significant disparity in in-hospital mortality between AIS patients with and without cardiac thrombus. However, we observed a notably higher 30-day readmission rate for hemorrhagic stroke and gastrointestinal bleeding in the cardiac thrombus group. These complications are likely attributable to anticoagulation therapy. Moreover, these patients may have been on dual antiplatelet therapy or even triple antithrombotic regimens if they had undergone percutaneous coronary intervention, which has been shown in previous studies to further elevate the risk of major bleeding events [8].

### Limitations

The results of this study should be interpreted in the context of several limitations. NRD is an administrative database that has the potential for miscoding. However, it is pertinent to mention here that NRD has a robust quality control program that minimizes such miscoding. The available data lacks imaging findings (echocardiography, CMR, CT) and details on cardiac thrombus size, all of which could impact clinical outcomes. The NRD also doesn’t capture out-of-hospital mortality rates.

## Conclusion

This study reveals that AIS patients with concurrent cardiac thrombus were more likely to undergo endovascular thrombectomy, possibly due to larger stroke size. An increased prevalence of cardiac thrombus in AIS hospitalizations was observed, potentially attributable to improved coding and increased cardiac CMR usage. While overall 30-day readmission rates were similar, patients with cardiac thrombus showed higher readmission rates for gastrointestinal bleeding and hemorrhagic stroke. Future research should focus on prospective studies to assess the impact of early cardiac thrombus detection on stroke management and outcomes. Investigation into optimal anticoagulation strategies and the mechanisms underlying increased bleeding risks in this population is also warranted. These efforts could inform targeted prevention strategies and improve clinical practice.

## Data Availability

No datasets were generated or analysed during the current study.

## References

[1] Kolominsky-Rabas PL, Weber M, Gefeller O, Neundoerfer B, Heuschmann PU. Epidemiology of ischemic stroke subtypes according to TOAST criteria: incidence, recurrence, and long-term survival in ischemic stroke subtypes: a population-based study. Stroke. 2001;32(12):2735–40. 11739965 10.1161/hs1201.100209

[2] O’Carroll CB, Barrett KM. Cardioembolic Stroke. Continuum (Minneap Minn). 2017;23(1, Cerebrovascular Disease):111–32. 28157747 10.1212/CON.0000000000000419

[3] Aljaber NN, Mattash ZA, Alshoabi SA, Alhazmi FH. The prevalence of left ventricular thrombus among patients with low ejection fraction by trans-thoracic echocardiography. Pak J Med Sci. 2020;36(4):673–7. 32494254 10.12669/pjms.36.4.1972 PMC7260930

[4] Inoue M, Noda R, Yamaguchi S, Tamai Y, Miyahara M, Yanagisawa S, et al. Specific factors to Predict large-vessel occlusion in Acute Stroke patients. J Stroke Cerebrovasc Dis. 2018;27(4):886–91. 29196201 10.1016/j.jstrokecerebrovasdis.2017.10.021

[5] Cost H, Project U. January. Overview of the nationwide readmissions database (NRD). Agency for Healthcare Research and Quality Accessed 2023;3.

[6] Chang P, Xiao J, Hu Z, Kwan AC, Fan Z. Imaging of left heart intracardiac thrombus: clinical needs, current imaging, and emerging cardiac magnetic resonance techniques. Ther Adv Cardiovasc Dis. 2022;16:17539447221107737. 35762763 10.1177/17539447221107737 PMC9243573

[7] Rinkel LA, Beemsterboer CF, Groeneveld NS, Lobe NH, Boekholdt SM, Bouma BJ, et al. Cardiac thrombi detected by CT in patients with acute ischemic stroke: a substudy of mind the heart. Eur Stroke J. 2023;8(1):168–74. 37021199 10.1177/23969873221130838 PMC10069221

[8] van Rein N, Heide-Jorgensen U, Lijfering WM, Dekkers OM, Sorensen HT, Cannegieter SC. Major bleeding rates in Atrial Fibrillation patients on single, dual, or Triple Antithrombotic Therapy. Circulation. 2019;139(6):775–86. 30586754 10.1161/CIRCULATIONAHA.118.036248

